# A Novel Linkable Ring Signature on Ideal Lattices

**DOI:** 10.3390/e25020237

**Published:** 2023-01-28

**Authors:** Chengtang Cao, Lin You, Gengran Hu

**Affiliations:** 1 School of Cyberspace Security, Hangzhou Dianzi University, Hangzhou 310018, China; 2 Department of Basic Science, Guizhou Industry Polytechnic College, Guiyang 550008, China

**Keywords:** ring signature, linkability, lattice

## Abstract

In this paper, a novel linkable ring signature scheme is constructed. The hash value of the public key in the ring and the signer’s private key are based on random numbers. This setting makes it unnecessary to set the linkable label separately for our constructed scheme. When judging the linkability, it is necessary to determine whether the number of the intersections of the two sets reaches the threshold related to the number of the ring members. In addition, under the random oracle model, the unforgeability is reduced to the $SVP_{\gamma }$ problem. The anonymity is proved based on the definition of statistical distance and its properties.

## 1. Introduction

In 2001, Rivest et al. [1] proposed the concept of ring signature. In a ring signature, the signer chooses several other users’ public keys to form a set with his own public key. In the signature verification phase, the verifier can confirm that the signature is generated by one of the ring members, but the verifier cannot find the real signer. There are many signature schemes that extend the original ring signature scheme to special scenarios, such as the deniable ring signature scheme in [2,3], the identity-based ring signature scheme in [4,5,6,7,8,9], and the linkable ring signature scheme in [10,11,12,13]. Linkable ring signature was a special ring signature proposed by Liu et al. [11]. Linkable ring signature is suitable for many practical scenarios, such as e-cash and e-voting. The general ring signature is not suitable for electronic voting because it is difficult to determine whether the same voter has voted more than once. Linkable ring signature can solve this problem, and the verifier can detect whether the generated votes are from the same voter through the linkable label. In 2021, Tang et al. [14] constructed an identity-based linkable ring signature scheme on NTRU lattice. In 2022, Ye et al. [15] constructed a linkable ring signature scheme on NTRU lattice. In [10,11,12,13,14,15], the linkability of the each signature scheme were determined by generating tags.

The signature schemes were based on the discrete logarithm in [1,11,13,16] and the bilinear pair in [17,18,19]. There are also parts of the literature that are based on lattices [3,14,20,21,22,23,24,25,26]. Lyubshvsky gave a signature scheme and a new hash function for calculating the difficulty problem based on ideal lattices in [27]. In [23], the first ring signature scheme was constructed by using the scheme [27]. In [3], a ring signature scheme with deniable property was constructed based on [3,27].

Based on [11,23,24], the output of the hash function of the public key in the ring and the signer’s private key are used to selecte random numbers. We give a new general structure of linkability, and construct a linkable ring signature scheme on ideal lattices (**LRS**).

### Contributions

• Replace the random number in the signature algorithm in [23] with the hash value of the public key in the ring and the private key. Our signature scheme (**LRS**) and the scheme in [23] have the same length of the public key, the secret key and the signature output, but our **LRS** is linkable.

• In [10,11,12,13,14,15,25,26], the linkable criterion was that the linkability label was the same. Unlike this, in our scheme, the linkability criterion is to determine the maximum number of the elements in the intersection of the two sets rather than the number of the ring members.

## 2. Preliminaries

### 2.1. Notations

The notations is in Table 1.

### 2.2. Hash Functions

**Definition** **1** ([28])**.** *For $m\in Z^{+}$ and $D_{h}\subseteq D$, let $H\left(D,D_{h},m\right)=\left\{h_{\hat{a}}:\hat{a}\in D^{m}\right\}$ be the function family such that for any $\hat{z}=\left(z_{1},z_{2},\ldots ,z_{m}\right)\in D_{h}^{m}$, $h_{\hat{a}}\left(\hat{z}\right)=\hat{a}\cdot \hat{z}=\Sigma_{i\in [m]}a_{i}z_{i}\in D$, where $\hat{a}=\left(a_{1},a_{2},\ldots ,a_{m}\right)$.*

According to [28], for $\hat{y},\hat{y}\in D_{h}^{m}$, c∈D and $h\in H(D,D_{h},m)$, then

$$\begin{array}{c} h\left(\hat{y}+{\hat{y}}^{\prime }\right)=h\left(\hat{y}\right)+h\left({\hat{y}}^{\prime }\right), \end{array}$$

$$\begin{array}{c} h\left(c\hat{y}\right)=c\cdot h\left(\hat{y}\right). \end{array}$$

**Definition** **2** ([28] Collision Problem)**.** *For $m\in Z^{+}$, $D_{h}\subseteq D$ and $h\in H(D,D_{h},m)$, the Collision Problem Col$\left(h,D_{h}\right)$ asks to find $\hat{y},{\hat{y}}^{\prime }\in D_{h}^{m}$ and $\hat{y}\neq {\hat{y}}^{\prime }$ such that $h\left(\hat{y}\right)=h\left({\hat{y}}^{\prime }\right)$.*

**Definition** **3** ([28])**.** *For γ>1, monic polynomial f and a lattice L corresponding to an ideal in the ring Z[x]/〈f〉, the $f-svp_{\gamma }$ problem asks to find g∈L such that ${∥g∥}_{\infty }\leq \gamma \lambda_{1}^{\infty }\left(L\right)$, where $\lambda_{1}$ is the length of the shortest nonzero vector on L.*

In Theorem 3.1 of the literature [27], if $f=x^{n}+1$ (where $n=2^{k},k\in Z^{+}$), we can get the following theorem.

**Theorem** **1** ([27])**.** *Let $D=Z_{p}\left[x\right]/\langle x^{n}+1\rangle$ be a ring (where $n=2^{k},k\in Z^{+}$). Define the set $D_{h}={\{y\in D:∥y∥}_{\infty }\leq d,\;d\in Z^{+}\}$. Let $H(D,D_{h},m)$ be a function family as in Definition 1 such that $m>\frac{\log p}{\log2d}$ and $p\geq 4dmn^{1.5}\log n$. If there is a polynomial-time algorithm that can solve Col$\left(h_{\hat{a}},D_{h}\right)$ for random $h_{\hat{a}}\in H\left(D,D_{h},m\right)$ with some non-negligible probability, then there is a polynomial-time algorithm that can solve $\left(x^{n}+1\right)-SVP_{\gamma }\left(L\right)$ for every lattice corresponding to an ideal in D, where $\gamma =16dmn\log^{2}n$.*

### 2.3. Statistical Distance

**Definition** **4** ([29])**.** *Let X and $X^{\prime }$ be two random variables over a countable set S. The statistical distance between X and $X^{\prime }$ is defined by*

$$\begin{array}{c} \Delta \left(X,X^{\prime }\right)=\frac{1}{2}\sum_{x\in S}\left|\mathrm{Pr}\left[X=x\right]-Pr\left[X^{\prime }=x\right]\right|. \end{array}$$

## 3. Framework and Security Model of LRS Scheme

Our **LRS** consists five probabilistic polynomial time (PPT) algorithms.

- SetUp: Input the security parameter *n*, and output the public parameter P.
- KeyGen: Input P, and output of a keypair (pk,sk).
- Sign: Input P, a singer’s (pk,sk), a message μ and the ring PK (pk∈PK), and output a signature σ.
- Verify: Input the signature σ, and output “1” or “0”.
- Link: Input two valid signatures $\left(\sigma_{1},\sigma_{2}\right)$, and output “1” or “0”.

The **LRS** is correct that the verification algorithm outputs “1” for the valid signature and “0” for the invalid signature.

### Security Properties

The **LRS** satisfies the unforgeabilityy, anonymit and linkability which is similar to [11,13,23].

**Definition** **5** (Unforgeability)**.** *The LRS is unforgeable if there is no PPT A to win the following games with an advantage that cannot be ignored.*

*Setup: C calls **LRS-SetUp** to generate the parameters P and calls **LRS-KeyGen** to generate the keypair $\left(pk_{i},sk_{i}\right)$, and sends the parameters P and all public keys $pk_{i}$ to A.*

*Query: the adversary A can perform polynomial Hash queries, Extract queries and Signature queries.*

Forgery: the adversary A submits $\left(i^{*},PK,\mu^{*},\sigma^{*}\right)$, if the following conditions are true:

*(1)*  A did not query the private key of $pk_{i^{*}}$;
*(2)*  A did not query $\left(pk_{i^{*}},\mu^{*}\right)$’s signature, then A won the game.

The advantage is defined as ${\mathrm{Adv}}_{A}^{\mathrm{forge}}=Pr\left[\mathrm{LRS}-\mathrm{Verify}\left(i^{*},PK,\mu^{*},\sigma^{*}\right)=1\right]$.

**Definition** **6** (Anonymity)**.** *The **LRS** scheme is said to be anonymous if there is no PPT A to win the following games with an advantage that cannot be ignored.*

*Setup: C calls **LRS-SetUp** to generate the parameters P and calls **LRS-KeyGen** to generate the keypair $\left(pk_{i},sk_{i}\right)$, and sends P and all public keys $pk_{i}$ to A.*

*Query: the A performs a polynomially bounded number of Hash queries, Extract queries and Signature queries.*

*Challenge: C selects b∈{0,1} and calls **LRS-Sign** $\left(b,PK,sk_{ib},\mu \right)$ (where PK, $sk_{ib}$ and μ are corresponding to the ring, the private key and the message respectively) to generate the signature $\sigma_{b,PK,sk_{ib},\mu }$. A did not query $\left(b,PK,sk_{ib},\mu \right)$’s signature.*

*Guess: A outputs $b^{\prime }$ as a guess of b. If $b^{\prime }=b$, then A wins the game.*

*The advantage is defined as ${\mathrm{Adv}}_{A}^{\mathrm{anon}}=\left|Pr\left[b^{\prime }=b\right]-\frac{1}{2}\right|$.*

**Definition** **7** (Linkability)**.** ***LRS** scheme is said to be linkable if for PPT A to win the following games with an advantage that cannot be ignored.*

*Setup: C calls **LRS-SetUp** to generate the parameters P and calls **LRS-KeyGen** to generate teh keypair $\left(pk_{i},sk_{i}\right)$, and sends P and all public keys $pk_{i}$ to A.*

*Query: the A performs a polynomially bounded number of Hash queries, Extract queries and Signature queries.*

*Challenge: C selects b∈{0,1} and calls **LRS-Sign** $\left(b,PK,sk_{ib},\mu \right)$ (where PK, $sk_{ib}$ and μ are corresponding to the ring, the private key and the message respectively) to generate the signature $\sigma_{b,PK,sk_{ib},\mu }$. A did not query $\left(b,PK,sk_{ib},\mu \right)$’s signature.*

*Guess: A outputs bit $b^{\prime }$ as a guess of b. If $b^{\prime }=b$ and $b^{\prime }\neq 1-b$, then A wins the game.*

*The advantage is defined as ${\mathrm{Adv}}_{A}^{\mathrm{link}}=\left|Pr\left[b^{\prime }=b\wedge b^{\prime }\neq 1-b\right]\right|$.*

## 4. Construction of Our LRS

The **LRS** consists of five PPT algorithms: ParamGen, KeyGen, Sign, Verify and Link. The parameter settings are as follows:

*D*: $\left\{f\in D:∥f{∥}_{\infty }\leq mn^{1.5}\log n+\sqrt{n}\log n\right\}$.

$D_{c}$: $\left\{f\in D:∥f{∥}_{\infty }\leq 1\right\}$.

$D_{y}$: $\left\{f\in D:∥f{∥}_{\infty }\leq mn^{1.5}\log n\right\}$.

*G*: $\left\{f\in D:∥f{∥}_{\infty }\leq mn^{1.5}\log n-\sqrt{n}\log n\right\}$.

*H*: ${\left\{0,1\right\}}^{*}\to G^{m}$.

$H_{1}$: ${\left\{0,1\right\}}^{*}\to D_{y}^{m}$.

$H_{2}$: ${\left\{0,1\right\}}^{*}\to D_{c}$.

H: a family of hash function: $D^{m}\to D$.

### 4.1. LRS-Setup

Step 1. Pick $k\in Z^{+}$.

Step 2. Pick $n=2^{\lambda }$, where $\lambda \in Z^{+}$ and λ>k. Let m=3logn.

Step 3. Pick *p* as a prime and $p>n^{4}$, p≡3mod8.

Step 4. Pick $h\overset{\$}{\leftarrow }H$.

Step 5. Output P=(k,n,m,h).

### 4.2. LRS-KeyGen

Step 1. Pick $\hat{s}\overset{\$}{\leftarrow }D_{c}^{m}$.

Step 2. Compute $P=h(\hat{s})$.

Step 3. Output $\left(pk,sk\right)=\left(P,\hat{s}\right)$.

### 4.3. LRS-Sign

Input a message μ, a ring $PK={\left\{P_{i}\right\}}_{i\in [l]}\subseteq D$, a private key ${\hat{s}}_{j}$ associated to the public key $P_{j}\in PK$, and do the following:

Step 1. For i∈[l]∖{j}, compute ${\hat{u}}_{i}=H\left(PK\setminus \left\{P_{i}\right\},{\hat{s}}_{j}\right)$.

Step 2. For i=j, compute ${\hat{u}}_{j}=H_{1}\left(PK\setminus \left\{P_{j}\right\},{\hat{s}}_{j}\right)$.

Step 3. Compute $R_{j}=h\left({\hat{u}}_{j}\right)$.

Step 4. Compute $c_{j+1}=H_{2}\left(\mu ,R_{j}\right)$.

Step 5. Compute

$$\begin{array}{ccc} & & R_{j+1}=h\left({\hat{u}}_{j+1}\right)-c_{j+1}\cdot P_{j+1}\\ & & c_{j+2}=H_{2}\left(\mu ,R_{j+1}\right)\\ & & R_{j+2}=h\left({\hat{u}}_{j+2}\right)-c_{j+2}\cdot P_{j+2}\\ & & c_{j+3}=H_{2}\left(\mu ,R_{j+2}\right)\\ & & \cdots \\ & & R_{l-1}=h\left({\hat{u}}_{l-1}\right)-c_{l-1}\cdot P_{l-1}\\ & & c_{l}=H_{2}\left(\mu ,R_{l-1}\right)\\ & & R_{l}=h\left({\hat{u}}_{1}\right)-c_{l}\cdot P_{l}\\ & & c_{1}=H_{2}\left(\mu ,R_{l}\right)\\ & & R_{1}=h\left({\hat{u}}_{1}\right)-c_{1}\cdot P_{l}\\ & & c_{2}=H_{2}\left(\mu ,R_{1}\right)\\ & & \cdots \\ & & R_{j-1}=h\left({\hat{u}}_{j-1}\right)-c_{j-1}\cdot P_{j-1}\\ & & c_{j}=H_{2}\left(\mu ,R_{j-1}\right) \end{array}$$

Step 6. For i=j, compute ${\hat{z}}_{j}={\hat{u}}_{j}+c_{j}\hat{s}$. If ${\hat{z}}_{j}\in D_{y}^{m}$ does not hold, then go back to reselect public keys.

Step 7. For i∈[l]∖{j}, ${\hat{z}}_{i}={\hat{u}}_{i}$.

Step 8. Output $\sigma =({\hat{z}}_{1},{\hat{z}}_{2},\ldots ,{\hat{z}}_{l},c_{l})$.

### 4.4. LRS-Verify

Input the message μ, the ring PK, the signature $\sigma =({\hat{z}}_{1},{\hat{z}}_{2},\ldots ,{\hat{z}}_{l},c_{l})$, and check the following steps:

Step 1. Compute

$$\begin{array}{ccc} & & R_{l}=h\left({\hat{z}}_{l}\right)-c_{l}\cdot P_{l}\\ & & c_{l+1}=H_{2}\left(\mu ,R_{l}\right)\\ & & R_{l+1}=h\left({\hat{z}}_{1}\right)-c_{l+1}\cdot P_{1}\\ & & c_{l+2}=H_{2}\left(\mu ,R_{l+1}\right)\\ & & \cdots \\ & & R_{2l-1}=h\left({\hat{z}}_{l-1}\right)-c_{2}\cdot P_{l-1}\\ & & c_{2l}=H_{2}\left(\mu ,R_{2l-1}\right) \end{array}$$

Step 2. If $c_{2l}=c_{l}$, then output “1”, otherwise output “0”.

### 4.5. LRS-Link

Input two valid signatures $\sigma_{0}=\left({\hat{z}}_{10},{\hat{z}}_{20},\ldots ,{\hat{z}}_{l0},c_{l0}\right)$, $\sigma_{1}=\left({\hat{z}}_{11},{\hat{z}}_{21},\ldots ,{\hat{z}}_{l1},c_{l1}\right)$ and do the following:

Step 1. If $|\left\{{\hat{z}}_{10},{\hat{z}}_{20},\ldots ,{\hat{z}}_{l0}\right\}\cap \left\{{\hat{z}}_{11},{\hat{z}}_{21},\ldots ,{\hat{z}}_{l1}\right\}|\geq l-1$ holds, then output “0”.

Step 2. Otherwise, output “1”.

### 4.6. LRS-Correctness

1. From Corollary 6.2 of [27], we obtain that the probability of ${\hat{z}}_{j}\in G^{m}$ is approximately 1/e;
2. We need to show $R_{j}=h\left({\hat{u}}_{j}\right)=h\left({\hat{z}}_{j}\right)-c_{j}\cdot P_{j}$. Since ${\hat{z}}_{j}={\hat{u}}_{j}+c_{j}\hat{s}$, we have $R_{j}=h\left({\hat{u}}_{j}\right)=h\left({\hat{z}}_{j}-c_{j}\hat{s}\right)=h\left({\hat{z}}_{j}\right)-c_{j}h\left(\hat{s}\right)=h\left({\hat{z}}_{j}\right)-c_{j}P_{j}$.

### 4.7. Construction of Our RS

By changing the first and second steps of the **LRS-Sign**, the following ring signature scheme (**RS**) can be obtained.

The parameter setting is the same as **LRS**

• **RS-Setup**

This part is the same as **LRS-Setup**.

• **RS-KeyGen**

This part is the same as **LRS-KeyGen**.

• **RS-Sign**

Input μ, a ring $PK={\left\{P_{i}\right\}}_{i\in [l]}\subseteq D$, a private key ${\hat{s}}_{j}$ associated to $P_{j}\in PK$, and do the following:

Step 1. For i∈[l]∖{j}, picks ${\hat{u}}_{i}\overset{\$}{\leftarrow }D_{y}^{m}$.

Step 2. For i=j, pick ${\hat{u}}_{j}\overset{\$}{\leftarrow }G^{m}$.

Step 3. Compute $R_{j}=h\left({\hat{u}}_{j}\right)$.

Step 4. Compute $c_{j+1}=H_{2}\left(\mu ,R_{j}\right)$.

Step 5. Compute

$$\begin{array}{ccc} & & R_{j+1}=h\left({\hat{u}}_{j+1}\right)-c_{j+1}\cdot P_{j+1}\\ & & c_{j+2}=H_{2}\left(\mu ,R_{j+1}\right)\\ & & R_{j+2}=h\left({\hat{u}}_{j+2}\right)-c_{j+2}\cdot P_{j+2}\\ & & c_{j+3}=H_{2}\left(\mu ,R_{j+2}\right)\\ & & \cdots \\ & & R_{l-1}=h\left({\hat{u}}_{l-1}\right)-c_{l-1}\cdot P_{l-1}\\ & & c_{l}=H_{2}\left(\mu ,R_{l-1}\right)\\ & & R_{l}=h\left({\hat{u}}_{1}\right)-c_{l}\cdot P_{l}\\ & & c_{1}=H_{2}\left(\mu ,R_{l}\right)\\ & & R_{1}=h\left({\hat{u}}_{1}\right)-c_{1}\cdot P_{l}\\ & & c_{2}=H_{2}\left(\mu ,R_{1}\right)\\ & & \cdots \\ & & R_{j-1}=h\left({\hat{u}}_{j-1}\right)-c_{j-1}\cdot P_{j-1}\\ & & c_{j}=H_{2}\left(\mu ,R_{j-1}\right) \end{array}$$

Step 6. For i=j, compute ${\hat{z}}_{j}={\hat{u}}_{j}+c_{j}\hat{s}$. If ${\hat{z}}_{j}\in D_{y}^{m}$ does not hold, then go back to reselect public keys.

Step 7. For i∈[l]∖{j}, ${\hat{z}}_{i}={\hat{u}}_{i}$

Step 8. Output $\sigma =({\hat{z}}_{1},{\hat{z}}_{2},\ldots ,{\hat{z}}_{l},c_{l})$.

•**RS-Vrify**

This part is the same as **LRS-Vrify**.

## 5. Security Analysis

We will prove that our **LRS** satisfies unforgeability, anonymity and linkability.

**Theorem** **2** (Unforgeability)**.** *If there is a PPT algorithm A which can forge the **LRS** signature with probabilistic ϵ at most q times random oracle H. Then for $h\overset{\$}{\leftarrow }H\left(D,m\right)$, there is a PPT algorithm B that outputs a solution to Col(h,D) with probability at least*

$$\begin{array}{c} \left(\epsilon -\frac{1}{|D_{c}|}\right)\left(\frac{\varepsilon -\frac{1}{|D_{c}|}}{q}-\frac{1}{|D_{c}|}\right)-\frac{1}{|D_{c}|}. \end{array}$$

**Proof** **of Theorem 2.** B gives an h∈H(D,m), picks a secret key $\hat{s}\overset{\$}{\leftarrow }D_{c}^{m}$ and computes the public key $P=h(\hat{s})$.

B creates two empty lists $L_{1},L_{2}$ to record the queries of adversary A.

Setup: Executing the **LRS-Setup**, B gives A the parameters P=(k,n,m,h).

Query: For the ring $PK={\left\{P_{i}\right\}}_{i\in [l]}\subseteq D$, where $P_{l}=P$, B performs the following operations:

Hash query:

1. A sends message μ to B. For i∈[l−1], B picks ${\hat{y}}_{i}\in D_{y}^{m}$ and ${\hat{y}}_{l}\in G^{m}$. B queries $L_{1}$ and returns the same record if there is already the query;
2. Otherwise, B picks $c_{l}\in D_{c}$ and passes $c_{l}$ to A. B records
  $$\left(\mu ,PK,\left({\hat{y}}_{1},{\hat{y}}_{2},\ldots ,{\hat{y}}_{l}\right),c_{l}\right)$$
  to $L_{1}$.

Extract query:

1. B queries $L_{2}$ first. If $\left(P_{l},{\hat{s}}_{i}\right)$ has already been queried, B returns $\left(P_{l},{\hat{s}}_{i}\right)$;
2. Otherwise, B picks ${\hat{s}}_{i}\in D_{c}^{m}$, and passes to A. B records $\left(P_{l},{\hat{s}}_{i}\right)$ to $L_{2}$.

Sign query:

A sends message μ, the ring $PK={\left\{P_{i}\right\}}_{i\in [l]}\subseteq D$, where $P_{l}=P$. B operates as follows:

1. B checks $L_{1}$. If $\left(\mu ,PK,\left({\hat{y}}_{1},{\hat{y}}_{2},\ldots ,{\hat{y}}_{l}\right),c_{l}\right)$ does not exist, go to Hash query and record $\left(\mu ,PK,\left({\hat{y}}_{1},{\hat{y}}_{2},\ldots ,{\hat{y}}_{l}\right),c_{l}\right)$ in $L_{1}$.
2. B checks $L_{2}$. If $\left(P_{i},{\hat{s}}_{i}\right)$ does not exist, go to Extract query and record $\left(P_{i},{\hat{s}}_{i}\right)$ in $L_{2}$.
3. B checks $L_{1}$ and $L_{2}$. B seeks the record $\left(\mu ,P,\left({\hat{y}}_{1},{\hat{y}}_{2},\ldots ,{\hat{y}}_{l}\right),c_{l}\right)$ in $L_{1}$ and the record $\left(P_{l},{\hat{s}}_{i}\right)$ in $L_{2}$;
4. Let ${\hat{z}}_{j}={\hat{y}}_{j}\left(j\neq l\right)$, ${\hat{z}}_{l}={\hat{y}}_{l}+c_{l}\hat{s}$, B returns the signature $\left({\hat{z}}_{1},{\hat{z}}_{2},\ldots ,{\hat{z}}_{l},c_{l}\right)$.

Forgery:

A sends a message $\mu^{*}$, the ring

$$PK^{*}=\left\{P_{i1^{*}},P_{i2^{*}},\ldots ,P_{il^{*}}\right\}\subseteq D$$

and forges signature $\left({\hat{z}}_{1}^{*},{\hat{z}}_{2}^{*},\ldots ,{\hat{z}}_{l}^{*},c_{l}^{*}\right)$ by the real signer $P_{il^{*}}$ to B, the following hold:

1. A has not inquired the private key of the public key $P_{il^{*}}$;
2. A has not inquired $\left(PK^{*},\mu^{*}\right)$’s signature.

Suppose the signature $\left({\hat{z}}_{1}^{*},{\hat{z}}_{2}^{*},\ldots ,{\hat{z}}_{l}^{*},c_{l}^{*}\right)$ is legal signature of message $\mu^{*}$ and $PK^{*}$. B first queries $L_{1}$ to find $\left(\mu^{*},PK^{*},\left({\hat{y}}_{1}^{*},{\hat{y}}_{2}^{*},\ldots ,{\hat{y}}_{l}^{*}\right),c_{l}^{*}\right)$ and queries $L_{2}$ to find $\left(P_{il^{*}},{\hat{s}}_{il^{*}}\right)$. If $\left(\mu^{*},PK^{*},\left({\hat{y}}_{1}^{*},{\hat{y}}_{2}^{*},\ldots ,{\hat{y}}_{l}^{*}\right),c_{l}^{*}\right)$ is not in $L_{1}$, the game ends. Otherwise, since $\left({\hat{z}}_{1}^{*},{\hat{z}}_{2}^{*},\ldots ,{\hat{z}}_{l}^{*},c_{l}^{*}\right)$ can pass the verification, we obtain

(1) $$\begin{array}{c} h\left({\hat{y}}_{l}^{*}\right)=h\left({\hat{z}}_{l}^{*}-c_{l}^{*}{\hat{s}}_{il^{*}}\right)=h\left({\hat{z}}_{l}^{*}\right)-c_{l}^{*}P_{il^{*}}. \end{array}$$

B answers A’s query again and answers all queries consistently except Hash returned by the $c_{l}$ query. By Lemma 3.1 in [30], A produces another forged signature $\left({\hat{z}}_{1}^{\prime },{\hat{z}}_{2}^{\prime },\ldots ,{\hat{z}}_{l}^{\prime },c_{l}^{\prime }\right)$, we obtain

(2) $$\begin{array}{c} h\left({\hat{y}}_{l}^{*}\right)=h\left({\hat{z}}_{l}^{\prime }-c_{l}^{\prime }{\hat{s}}_{il^{*}}\right)=h\left({\hat{z}}_{l}^{\prime }\right)-c_{l}^{\prime }P_{il^{*}}. \end{array}$$

From (1) and (2), we obtain $h\left({\hat{z}}_{l}^{*}-c_{l}^{*}{\hat{s}}_{il^{*}}\right)=h\left({\hat{z}}_{l}^{\prime }-c_{l}^{\prime }{\hat{s}}_{il^{*}}\right)$. If ${\hat{z}}_{l}^{*}-c_{l}^{*}{\hat{s}}_{il^{*}}\neq {\hat{z}}_{l}^{\prime }-c_{l}^{\prime }{\hat{s}}_{il^{*}}$, since ${\hat{z}}_{l}^{*}-c_{l}^{*}{\hat{s}}_{il^{*}},{\hat{z}}_{l}^{\prime }-c_{l}^{\prime }{\hat{s}}_{il^{*}}\in D_{y}^{m}$, we solved the problem Col(h,D).

B extracts the secret key $\hat{s}$ of $P_{l}$, and lets $z_{i}^{\prime \prime }={\hat{y}}_{i}^{*}$ (if i≠l), ${\hat{z}}_{l}^{\prime \prime }={\hat{y}}_{l}^{*}+c_{l}\hat{s}$. It is easy to see that $\left({\hat{z}}_{1}^{\prime \prime },{\hat{z}}_{2}^{\prime \prime },\cdots ,{\hat{z}}_{l}^{\prime \prime },c_{l}\right)$ can pass the verification, so

(3) $$\begin{array}{c} h\left({\hat{y}}_{l}^{*}\right)=h\left({\hat{z}}_{l}^{\prime \prime }-c_{l}^{*}\hat{s}\right)=h\left({\hat{z}}_{l}^{\prime \prime }\right)-c_{l}^{*}P_{il^{*}}. \end{array}$$

B continues the calculation. Let $z_{i}^{\prime \prime \prime }={\hat{y}}_{i}^{*}$ (if i≠l), ${\hat{z}}_{l}^{\prime \prime \prime }={\hat{y}}_{l}^{*}+c_{l}^{\prime }\hat{s}$. We will obtain $\left({\hat{z}}_{1}^{\prime \prime \prime },{\hat{z}}_{2}^{\prime \prime \prime },\ldots ,{\hat{z}}_{l}^{\prime \prime \prime },c_{l}^{\prime }\right)$ can pass the verification, so

(4) $$\begin{array}{c} h\left({\hat{y}}_{l}^{*}\right)=h\left({\hat{z}}_{l}^{\prime \prime \prime }-c_{l}^{\prime }\hat{s}\right)=h\left({\hat{z}}_{l}^{\prime \prime \prime }\right)-c_{l}^{\prime }P_{il^{*}}. \end{array}$$

From (1) and (3), we obtain $h\left({\hat{z}}_{l}^{*}\right)=h\left({\hat{z}}_{l}^{\prime \prime }\right)$. If ${\hat{z}}_{l}^{*}\neq {\hat{z}}_{l}^{\prime \prime }$, since ${\hat{z}}_{l}^{*},{\hat{z}}_{l}^{\prime \prime }\in G^{m}$, we solved the problem Col(h,D).

From (2) and (4), we obtain $h\left({\hat{z}}_{l}^{\prime }\right)=h\left({\hat{z}}_{l}^{\prime \prime \prime }\right)$. If ${\hat{z}}_{l}^{\prime }\neq {\hat{z}}_{l}^{\prime \prime \prime }$, since ${\hat{z}}_{l}^{\prime },{\hat{z}}_{l}^{\prime \prime \prime }\in G^{m}$, we also solved the problem Col(h,D).

If ${\hat{z}}_{l}^{*}-c_{l}^{*}{\hat{s}}_{il^{*}}={\hat{z}}_{l}^{\prime }-c_{l}^{\prime }{\hat{s}}_{il^{*}}$, ${\hat{z}}_{l}^{*}={\hat{z}}_{l}^{\prime \prime }$, ${\hat{z}}_{l}^{\prime }={\hat{z}}_{l}^{\prime \prime \prime }$, from (3) and (4), we obtain $h\left({\hat{z}}_{l}^{*}-c_{l}^{*}\hat{s}\right)=h\left({\hat{z}}_{l}^{\prime }-c_{l}^{\prime }\hat{s}\right)$. As discussed in Theorem 6.6 in [27], we can get ${\hat{z}}_{l}^{*}-c_{l}^{*}\hat{s}\neq {\hat{z}}_{l}^{\prime }-c_{l}^{\prime }\hat{s}$.

If

(5) $$\begin{array}{c} {\hat{z}}_{l}^{*}-c_{l}^{*}\hat{s}={\hat{z}}_{l}^{\prime }-c_{l}^{\prime }\hat{s}, \end{array}$$

from (5) and ${\hat{z}}_{l}^{*}-c_{l}^{*}{\hat{s}}_{il^{*}}={\hat{z}}_{l}^{\prime }-c_{l}^{\prime }{\hat{s}}_{il^{*}}$, we obtain

$$\begin{array}{c} \left({\hat{s}}_{il^{*}}-\hat{s}\right)\left(c_{l}^{*}-c_{l}^{\prime }\right)=0. \end{array}$$

Since $∥{\hat{s}}_{il^{*}}{∥}_{\infty }\leq 1,∥\hat{s}{∥}_{\infty }\leq 1,∥c_{l}^{*}{∥}_{\infty }\leq 1,{∥c_{l}^{\prime }∥}_{\infty }\leq 1$, we obtain $∥{\hat{s}}_{il^{*}}-\hat{s}{∥}_{\infty }\leq 2,{∥c_{l}^{*}-c_{l}^{\prime }∥}_{\infty }\leq 2$, so $∥\left({\hat{s}}_{il^{*}}-\hat{s}\right)\left(c_{l}^{*}-c_{l}^{\prime }\right){∥}_{\infty }\leq 4n$.

Since $p>n^{4}$, $4n\leq \frac{p}{2}$, so the product $\left({\hat{s}}_{il^{*}}-\hat{s}\right)\left(c_{l}^{*}-c_{l}^{\prime }\right)$ is 0 in the ring $Z_{p}\left[x\right]/\langle x^{n}+1\rangle$, it also must be 0 in the ring $Z_{p}\left[x\right]/\langle x^{n}+1\rangle$. Because $x^{n}+1$ is irreducible over the integers, $Z_{p}\left[x\right]/\langle x^{n}+1\rangle$ is an integral domain, therefore either ${\hat{s}}_{il^{*}}-\hat{s}=0$ or $c_{l}^{*}-c_{l}^{\prime }=0$. Since $c_{l}^{*}\neq c_{l}^{\prime }$ and ${\hat{s}}_{il^{*}}\neq \hat{s}$, so

$$\begin{array}{c} {\hat{z}}_{l}^{*}-c_{l}^{*}\hat{s}\neq {\hat{z}}_{l}^{\prime }-c_{l}^{\prime }\hat{s}. \end{array}$$

Thus the problem Col(h,D) was solved.

Suppose the probability that B can successfully solve Col(h,D) is $\epsilon^{\prime }$.

When $\left({\hat{z}}_{1}^{*},{\hat{z}}_{2}^{*},\ldots ,{\hat{z}}_{l}^{*},c_{l}^{*}\right)$ is not in $L_{1}$, the probability that $c_{l}^{*}$ passing the **LRS-verify** is $\frac{1}{|D_{c}|}$.

By Lemma 3.1 in [30], we get that the probability of Equation (2) is

$$\begin{array}{c} \left(\epsilon -\frac{1}{|D_{c}|}\right)\left(\frac{\varepsilon -\frac{1}{|D_{c}|}}{q}-\frac{1}{|D_{c}|}\right). \end{array}$$

From the above analysis, we can see that

$$\begin{array}{c} \varepsilon^{\prime }\geq \left(\epsilon -\frac{1}{|D_{c}|}\right)\left(\frac{\varepsilon -\frac{1}{|D_{c}|}}{q}-\frac{1}{|D_{c}|}\right)-\frac{1}{|D_{c}|}. \end{array}$$

From Theorem 1, we obtain that Col(h,D) is based on solving $\left(x^{n}+1\right)-Svp_{\gamma }\left(L\right)$ (where $\gamma =\tilde{O}\left(n^{3}\right))$ for every lattice L corresponding to an ideal D. □

**Theorem** **3** (Anonymity)**.** *For b∈{0,1}, $\sigma_{b,PK,sk_{ib},\mu }$ are the outputs of the algorithm **LRS-Sign** $\left(b,PK,sk_{ib},\mu \right)$, where PK, $sk_{ib}$ and μ are corresponding to the ring, the private key and the message respectively. For any PPT adversary, when $sk_{i0}$ and $sk_{i1}$ are unknown, then*

$$\begin{array}{c} \Delta (\sigma_{0,P,sk_{ib},\mu },\sigma_{1,P,sk_{ib},\mu })=0. \end{array}$$

*Therefore, **LRS** is anonymous.*

**Proof** **of Theorem 3.** Setup: This part is the same as in Theorem 2.

Query: This part is the same as Theorem 2.

Challenge: C selects the message μ, keypair $\left(pk_{ib},sk_{ib}\right)$, the ring PK and $pk_{ib}\in PK$, then randomly selects b∈{0,1} and calls **LRS-Sign**$\left(b,PK,sk_{ib},\mu \right)$ to generate the signature $\sigma_{b,PK,sk_{ib},\mu }$.

Guess: A outputs $b^{\prime }$.

Suppose the signature with private key $sk_{i0}$ outputs

$$\sigma_{0,P,sk_{i0},\mu }=\left({\hat{z}}_{10},{\hat{z}}_{20},\ldots ,{\hat{z}}_{l0},c_{l0}\right),$$

the signature with private key $sk_{i1}$ outputs

$$\sigma_{1,P,sk_{i1},\mu }=\left({\hat{z}}_{11},{\hat{z}}_{21},\ldots ,{\hat{z}}_{l1},c_{l1}\right).$$

The following only need to prove that $\sigma_{0,P,sk_{i0},\mu }$ and $\sigma_{1,P,sk_{i1},\mu }$ are statistically indistinguishable.

From Proposition 8.9, 8.10 of [29] and trigonometric inequality, we can get

$$\begin{array}{cc} \Delta (\sigma_{0,P,sk_{i0},\mu },\sigma_{1,P,sk_{i1},\mu }) & =\Delta (\left({\hat{z}}_{10},{\hat{z}}_{20},\ldots ,{\hat{z}}_{l0},c_{l0}\right),\left({\hat{z}}_{11},{\hat{z}}_{21},\ldots ,{\hat{z}}_{l1},c_{l1}\right))\\ \; & \leq \Delta (\left(P_{1},P_{2},\ldots ,P_{l},sk_{i0}\right),\left(P_{1},P_{2},\ldots ,P_{l},sk_{i1}\right))\\ \; & \leq \Delta (sk_{i0},sk_{i1})\\ \; & =\frac{1}{2}\sum_{\hat{a}\in D_{c}^{m}}\left|Pr\left[sk_{i0}=\hat{a}\right]-Pr\left[sk_{i1}=\hat{a}\right]\right|\\ \; & =\frac{1}{2}\sum_{\hat{a}\in D_{c}^{m}}\left|\frac{1}{|D_{c}^{m}|}-\frac{1}{|D_{c}^{m}|}\right|\\ \; & =0. \end{array}$$

□

**Theorem** **4** (Linkability)**.** *If H is collision resistant and the number of ring members is not less than three, then the **LRS** signature scheme is linkable.*

**Proof** **of Theorem 4.** Setup: This part is the same as in Theorem 2.

Query: This part is the same as Theorem 2.

Challenge:

1. C hands a message μ and uses the **LRS-KeyGen** to generate key pair
  $$\left(P_{k0},{\hat{s}}_{k0}\right),\left(P_{k1},{\hat{s}}_{k1}\right).$$
2. C picks the ring $PK={\left\{P_{i}\right\}}_{i\in [l]}$ and $\{P_{k0},P_{k1}\}\subseteq PK$. C calls **LRS-Sign** to generate the signatures $\sigma_{0}=\left({\hat{z}}_{10},{\hat{z}}_{20},\ldots ,{\hat{z}}_{l0},c_{l0}\right)$ and $\sigma_{1}=\left({\hat{z}}_{11},{\hat{z}}_{21},\ldots ,{\hat{z}}_{l1},c_{l1}\right)$.
3. C picks b∈{0,1}, then reselects $\mu^{*}$ and uses the ring $PK={\left\{P_{i}\right\}}_{i\in [l]}$ to call the **LRS-KeyGen** to generate the signature $\sigma_{b}^{\prime }=\left({\hat{z}}_{1b}^{\prime },{\hat{z}}_{2b}^{\prime },\ldots ,{\hat{z}}_{lb}^{\prime },c_{lb}^{\prime }\right)$. C sends $\sigma_{b}^{\prime }$ to A.

Guess: A outputs bit $b^{\prime }$.

A decides which of

$$|\left\{{\hat{z}}_{10},{\hat{z}}_{20},\ldots ,{\hat{z}}_{l0}\right\}\cap \left\{{\hat{z}}_{1b}^{\prime },{\hat{z}}_{2b}^{\prime },\ldots ,{\hat{z}}_{lb}^{\prime }\right\}|\geq l-1$$

and

$$|\left\{{\hat{z}}_{11},{\hat{z}}_{21},\ldots ,{\hat{z}}_{l1}\right\}\cap \left\{{\hat{z}}_{1b}^{\prime },{\hat{z}}_{2b}^{\prime },\ldots ,{\hat{z}}_{lb}^{\prime }\right\}|\geq l-1$$

holds. If the first is true, output $b^{\prime }=0$, if the second is true, output $b^{\prime }=1$.

Next, we will discuss it in two ways.

1. When ${\hat{s}}_{kb}={\hat{s}}_{k0}$, because the ring $PK={\left\{P_{i}\right\}}_{i\in [l]}$ is the same and the calculated ${\hat{u}}_{i}$ is the same, there is at most one output ${\hat{z}}_{i}$ of the signature output which is different from the real signer’s subscript, so there are identical ${\hat{z}}_{i}$ at least l−1. That is, when the signature is signed by the same private key for different messages, it can be completely determined.
2. when ${\hat{s}}_{kb}\neq {\hat{s}}_{k0}$, because the ring $PK={\left\{P_{i}\right\}}_{i\in [l]}$ is the same and *H* is strong anti-collision, when calculating ${\hat{u}}_{i}=H\left(PK\setminus \left\{P_{i}\right\},{\hat{s}}_{j}\right)$, the probability that the hash values ${\hat{u}}_{i}=H\left(PK\setminus \left\{P_{i}\right\},{\hat{s}}_{kb}\right)$ and ${\hat{u}}_{i}=H\left(PK\setminus P_{i},{\hat{s}}_{k0}\right)$ are equal can be negligible. Therefore, only one probability is negligible at most with the same output value as the real signer subscript.

Since there are at least three ring members and at least two ${\hat{z}}_{i}$’s are not the same, when the signature is not the same signer, it can be determined with overwhelming probability.

□

## 6. Efficiency Analysis

In Table 2, we set $\theta =mn^{1.5}\log n-\sqrt{n}\log n$ and *l* is the number of ring members. From Table 2, we may conclude that the public key, secret key and signature sizes of our scheme are equal to the scheme in [23], the size of the signature is smaller than the scheme in [3], and the size of the signature is larger than the scheme in [15].

In Table 3, *m* is the number of components of a polynomial vector and *l* is the number of ring members. When calculating the time complexity, some lightweight operations (hash function and random number selecting) are not taken into account. It mainly calculates the time cost of polynomial multiplication ($T_{Mul}$) and polynomial inversion ($T_{Inv}$). The runtime of the discrete Gaussian sampling algorithm, the rejection sampling algorithm, the trapdoor generation algorithm and the SamplePre algorithm [15] are represented by $T_{Sd}$, $T_{Rs}$, $T_{Trap}$ and $T_{Sam}$, respectively. In [15], $T_{Trap}$, $T_{Sam}$, $T_{Sd}$ and $T_{Rs}$ are used for keypair and the signature. From Table 3, we may conclude that the signature cost and the verification cost in our scheme are smaller than the scheme in [3], and the keypair cost is smaller than the scheme in [3,23].

Table 4 shows the comparison of our signature scheme with the other four schemes in terms of their functionality. The deniable ring signature can prove that the ring member has not signed the signature when necessary. The linkable ring signature can determine whether two signatures are those of the same signer in the ring member. Both the deniable ring signature and the linkable ring signature are ring signatures with special properties, which can be applied to special real situations. From Table 4, we may conclude that **LRS** and YQ [15] are linkable and secure in case of a quantum attack.

## 7. Conclusions

In this paper, the **LRS** is constructed based on the $SVP_{\gamma }\left(L\right)$ problem. In **LRS**, the linkable label is embedded into the randomly selected vector of the signature process in the constructed signature scheme in [23], which means that although the signature output form of our scheme is the same as in the scheme in [23], our scheme is linkable. In the future, we hope to construct a linkable and deniable ring signature scheme. 

## Figures and Tables

**Table 1:** Notations.

Symbol	Description
|S|	If $S=\{s_{1},s_{2},\ldots ,s_{n}\}$, then |S|=n.
[i]	{1,2,…,i}.
$x\overset{\$}{\leftarrow }S$	*x* is a uniformly random sample from the set *S*.
$Z_{p}$	Z/pZ.
D	$\left\{a\in Z_{p}\left[x\right]/\langle x^{n}+1\rangle :a=\sum_{i=0}^{n-1}a_{i}x^{i},a_{i}\in \left\{-\frac{p-1}{2},\ldots ,\frac{p-1}{2}\right\}\right\}$.
L	the ideal lattice.
${∥a∥}_{\infty }$	${∥a∥}_{\infty }=max_{i}\left(a_{i}\right)$, where $a=\sum_{i=0}^{n}a_{i}x^{i}\in Z\left[x\right]$.
$\hat{a}$	$\hat{a}=\left(a_{1},a_{2},\ldots ,a_{m}\right)\in {\left(Z\left[x\right]\right)}^{m}$.
$∥\hat{a}{∥}_{\infty }$	$∥\hat{a}{∥}_{\infty }=max_{i}{∥a_{i}∥}_{\infty }$, where $a_{i}\in Z\left[x\right]$.

**Table 2:** Communication overhead comparison (in bits).

Scheme	Public Key	Secret Key	Signature
GW [3]	mnlogp	2mnlogp	2mnlogθ+2ln+(m+1)nlogp
AM [15]	nlogp	2nlogp	nllogp
AM [23]	mnlogp	2mnlogp	2mnlogθ+2n
**RS**	mnlogp	2mnlogp	2mnlogθ+2n
**LRS**	mnlogp	2mnlogp	2mnlogθ+2n

**Table 3:** Comparison of time costs.

Scheme	Keypair	Signature	Verification
GW [3]	$\left(2m-1\right)T_{Mul}+T_{Inv}$	$\left(2l+4m+3l-2\right)T_{Mul}$	$\left(2lm+3\right)T_{Mul}$
YQ [15]	$T_{Mul}+T_{Trap}+T_{Sam}$	$lT_{Mul}+2T_{Sd}+2T_{Rs}$	$lT_{Mul}$
AM [23]	$\left(2m-1\right)T_{Mul}+T_{Inv}$	$\left(lm+m\right)T_{Mul}$	$\left(lm+1\right)T_{Mul}$
**RS**	$mT_{Mul}$	$\left(lm+m+l-1\right)T_{Mul}$	$\left(lm+l\right)T_{Mul}$
**LRS**	$mT_{Mul}$	$\left(lm+m+l-1\right)T_{Mul}$	$\left(lm+l\right)T_{Mul}$

**Table 4:** Comparison of functionality.

Scheme	Quantum-Resistance	Deniability	Linkability
LJ [11]	No	No	Yes
GW [3]	Yes	Yes	No
YQ [15]	Yes	No	Yes
AM [23]	Yes	No	No
**RS**	Yes	No	No
**LRS**	Yes	No	Yes

## Data Availability

Not applicable.
